# Short interbirth interval and associated factors among women with antecedent cesarean deliveries at a tertiary hospital, Southwestern Uganda

**DOI:** 10.1186/s12884-022-04611-4

**Published:** 2022-03-30

**Authors:** Onesmus Byamukama, Richard Migisha, Paul Kato Kalyebara, Leevan Tibaijuka, Henry Mark Lugobe, Joseph Ngonzi, Onesmus Magezi Ahabwe, Kenia Raquel Martinez Garcia, Godfrey R. Mugyenyi, Adeline Adwoa Boatin, Joy Muhumuza, Wasswa G. M. Ssalongo, Musa Kayondo, Hamson Kanyesigye

**Affiliations:** 1 Department of Obstetrics and Gynecology, Faculty of Medicine, Mbarara University of Science and Technology, P.O. Box 1410, Mbarara, Uganda; 2 Department of Physiology, Faculty of Medicine, Mbarara University of Science and Technology, Mbarara, Uganda; 3 Department of Obstetrics and Gynecology, Mbarara Regional Referral Hospital, Mbarara, Uganda; 4 Department of Obstetrics and Gynecology, Massachusetts General Hospital, Boston, USA

**Keywords:** Birth spacing, Cesarean delivery, Birth Interval, Short birth interval, Uganda

## Abstract

**Background:**

Women with previous cesarean deliveries, have a heightened risk of poor maternal and perinatal outcomes, associated with short interbirth intervals. We determined the prevalence of short interbirth interval, and associated factors, among women with antecedent cesarean deliveries who delivered at Mbarara Regional Referral Hospital (MRRH), in southwestern Uganda.

**Methods:**

We conducted a cross-sectional study on the postnatal ward of MRRH from November 2020 to February 2021. We enrolled women who had antecedent cesarean deliveries through consecutive sampling. We obtained participants’ socio-demographic and obstetric characteristics through interviewer-administered structured questionnaires. We defined short interbirth interval as an interval between two successive births of < 33 months. Modified Poisson regression was used to identify factors associated with short interbirth intervals.

**Results:**

Of 440 participants enrolled, most had used postpartum family planning (PPFP) prior to the current pregnancy (67.5%), and most of the pregnancies (57.2%) were planned. The mean age of the participants was 27.6 ± 5.0 years. Of the 440 women, 147 had a short interbirth interval, for a prevalence of 33% (95%CI: 29–38%). In multivariable analysis, non-use of PPFP (adjusted prevalence ratio [aPR] = 2.24; 95%CI: 1.57–3.20, *P* < 0.001), delivery of a still birth at an antecedent delivery (aPR = 3.95; 95%CI: 1.43–10.9, *P* = 0.008), unplanned pregnancy (aPR = 3.59; 95%CI: 2.35–5.49, *P* < 0.001), and young maternal age (aPR = 0.25 for < 20 years vs 20–34 years; 95%CI: 0.10–0.64, *P* = 0.004), were the factors significantly associated with short interbirth interval.

**Conclusion:**

One out of every three womenwith antecedent caesarean delivery had a short interbirth interval. Short interbirth intervals were more common among women with history of still births, those who did not use postpartum family planning methods, and those whose pregnancies were unplanned, compared to their counterparts. Young mothers (< 20 years) were less likely to have short interbirth intervals compared to those who were 20 years or older. Efforts should be made to strengthen and scale up child-spacing programs targeting women with previous cesarean deliveries, given the high frequency of short interbirth intervals in this study population.

## Introduction

The World Health Organization (WHO) recommends an inter-pregnancy interval (birth-to-pregnancy interval) of not less than 24 months or a minimum interbirth interval of 33 months [1], in order to minimize perinatal mortality and improve maternal health. This recommendation is in line with the WHO's recommendation of a two-year minimum breastfeeding period [1]. Birth spacing patterns and practices vary worldwide, with women in low-income countries reported to have shorter interbirth intervals than their counterparts in high-income countries [2]. Globally, approximately 25% of births still occur at intervals less than the WHO recommendation; in sub-Saharan Africa, the prevalence of short birth interval is reported to be highest in Chad (30.18%) and the Democratic Republic of Congo (27.12%) [3, 4]. In Uganda, 24% of children are born less than two years after their siblings, with an estimated median birth interval of 32 months [5]. In southwestern Uganda, birth intervals haven’t previously been assessed.

Several factors, including maternal age, failure or lack of contraceptive use, family size, level of male partner involvement, and sex of the previous child have been reported to influence birth spacing [2, 6]. Nevertheless, short interbirth intervals are associated with an increased risk of cesarean section delivery, preterm births, small-for-gestational age babies, postpartum haemorrhage, ruptured uterus, and death [7]. In women with previous cesarean delivery the risk for these adverse outcomes may be amplified two to three fold [8].

Uganda has a high fertility rate of 5.4 children per woman [5]. Furthermore, Children born less than 24 months after a previous birth were reported to have a much higher rate of under-5 mortality (104 deaths per 1,000 live births) than children born three years or more after a previous birth (54 deaths per 1,000 live births) [5].

Few studies that have assessed interbirth intervals in Uganda have been conducted in the general population of women of child bearing age and yet women with previous cesarean deliveries have a two to three heightened risk in poor maternal and perinatal outcomes [6, 9]. Cesarean delivery rates vary widely across health facilities in Uganda, with regional referral hospitals more likely to have caesarean delivery rates > 30% compared to lower health facilities [10]. Cesarean delivery rates have increased both at health facility and population levels in Uganda. Overall, the cesarean section rate for live births at health facilities increased from 8.5% in 2012 to 11% in 2016; the overall population-based cesarean section rate was 4.7%, and increased from 3.2 to 5.9% over the same period [11]. Given the higher risk of unfavorable maternal and perinatal outcomes among women who have had cesarean section deliveries, it is critical to identify those who are likely to have short interbirth intervals after cesarean deliveries, in order to plan interventions tailored for them. Mbarara Regional Referral Hospital (MRRH), in particular, has been reported to have high cesarean delivery rates of > 25% [12]. This study determined the prevalence and associated factors of short interbirth interval among women with antecedent cesarean deliveries who delivered at MRRH in southwestern Uganda, to inform designing of evidence-based interventions aimed at improving birth spacing in the region.

## Methods

### Study setting, study design and study population

This was a cross-sectional study, conducted at the post-natal ward of Mbarara Regional Referral Hospital (MRRH) from November 11, 2020 to February 12, 2021. MRRH is located in southwestern Uganda, Mbarara City. The hospital serves as a regional referral hospital for southwestern Uganda, and also teaching hospital for Mbarara University Science and Technology (MUST). It has a total bed capacity of 350 beds, 40 of which are on postnatal ward. Every year, the department performs roughly 11,000 deliveries, of which 5,000 are cesarean deliveries, representing a caesarean delivery rate of 45%. Repeat cesarean sections account for about 2,000 of these cesarean section deliveries.

Our study population was postpartum mothers who delivered at MRRH, and whose previous delivery was by cesarean section. We excluded mothers who were unable to consent or give information about previous pregnancy, including those with altered level of consciousness.

### Sample size and sampling

We used Epi Info version 7.2 (CDC, Atlanta, USA) to estimate the sample size with the following assumptions: an arbitrary expected frequency of short interbirth interval of 52% [6], margin of error of 5%, at 95% level of confidence, from an estimated source population of 5,000 women with caesarean section deliveries. After factoring in a non-response rate of 20%, we estimated a sample size of 428 women. We enrolled the mothers on the postnatal ward who met the inclusion criteria into the study through consecutive sampling. Written informed consent was obtained from each study participant before recruitment and participation in the study.

### Data collection and study variables

Data were collected by two research assistants using an interviewer-administered structured questionnaires. The research assistants were midwives, who were trained on the data collection tool and study procedures.

Our outcome variable was interbirth interval which was dichotomized as short and non-short interbirth interval. We defined short interbirth interval as an interval between two successive births of less than 33 months regardless of the outcome of the antecedent birth [1].

The questionnaire captured data on independent variables including socio-demographic and obstetric factors. The socio-demographic characteristics included age, marital status, residency, occupation, religion, education level, and partner support. We categorised age to take into consideration the extremes of age as follows: < 20 years for teenagers, and advanced maternal age for those > 34 years. We considered partners to be supportive if they fulfilled any two of the following tasks: providing finances, making family planning decisions together and escorting the mother to the health facility. Obstetric factors included parity, previous pregnancy outcome (whether still birth or live birth), desire for fertility, preferred sex of the baby, sex of the baby at the antecedent pregnancy, breastfeeding, postnatal care attendance for the previous pregnancy, and resumption of menses. Data on use of postpartum family planning (PPFP), methods used and time of initiation of a postpartum family planning method were also obtained. We defined PPFP as the initiation and use of family planning services within the first 12 months following childbirth [13]. We categorized return of menses as < 6 months and ≥ 6 months after; this is because return of menses between birth and 6 months would indicate early return of menses especially for the women who are not exclusively breastfeeding [14]. We categorized resumption of coitus as < 1 month and ≥ 1 month postpartum; this is because initiation of coitus < 1 months postpartum is considered early resumption [15].

### Data management and analysis

Data were entered into Redcap and exported to STATA version 15 (*StataCorp*, College Station, Texas, USA) for analysis.

We described the demographic and obstetric characteristics of the study participants, and expressed the descriptive statistics as frequencies/percentages. We then compared the categorical variables between women with short interbirth interval and those with non-short interbirth interval, using Pearson’s chi square test. The prevalence of short interbirth interval was calculated as a proportion of women who met the definition of short interbirth interval— by dividing the number of women with short interbirth interval by the total number of postpartum women with antecedent cesarean deliveries, and expressed as a percentage.

To identify factors associated with short interbirth interval, we used modified Poisson regression model that included robust standard errors, based on a generalized linear model with the Poisson family and a log link without an offset. Corresponding prevalence ratios (PRs) with their 95% confidence intervals (CIs) were reported as our measures of association. In this cross-sectional study, the modified Poisson regression was chosen over logistic regression to avoid odds ratios overestimating the effect size, in our scenario where the prevalence of the outcome was high [16]. All variables associated with short interbirth interval at univariable analysis (with *P* value < 0.2) were included into the final multivariable model to determine the adjusted correlates of short interbirth interval. We assessed for collinearity using variance inflation factor (VIF); we eliminated highly correlated variable (with VIF > 5) in the final multivariable model. Variables with *P* values < 0.05 in the final model were considered statistically significant.

## Results

**Fig. 1:**
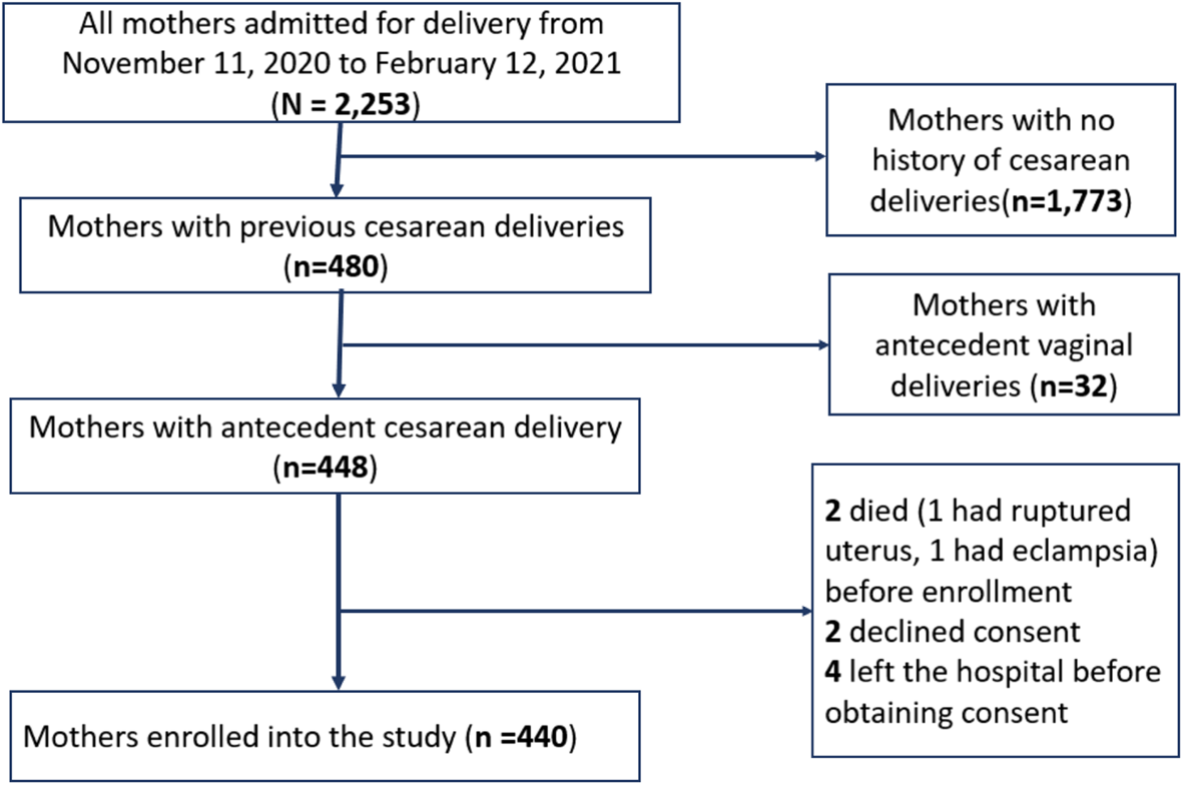
Flow chart for recruitment of study participants at Mbarara Regional Referral Hospital, southwestern Uganda, November 2020–February 2021

Out of 2,253 mothers admitted for delivery from November 2020 to February 2021 at MRRH, we present results for 440 participants who were enrolled into the study. Eight women, including four who left hospital before consenting, and two who declined to consent, were excluded from the study (Fig. 1).

### Socio-demographic characteristics of study participants

The mean age of the study participants was 27.6 ± 5.0 years. Of the 440 participants, most were of rural residence (58.4%), had attained primary education (42.5%), and were unemployed (62.9%); the majority (95.4%) were married. The proportions of unemployed women (78.1% Vs 58.4%; *P* = 0.005), and those residing in rural areas (66.7% Vs 54.3%; *P* = *0*.013) were significantly higher in the short interbirth interval group than in the non-short interbirth interval group (Table 1). Participants with short interbirth interval were of significantly younger age compared those without short interbirth interval (*P* = 0.010). Other socio-demographic characteristics were similar between the two groups.

**Table 1:** Socio-demographic characteristics of women with antecedent caesarean delivery at Mbarara Regional Referral Hospital

Variable	Total (*N* = 440) n (%)	Short inter-delivery interval, n (%)	
		Yes (*n* = 147)	No (*n* = 293)
**Age in years**			
< 20	46 (10.5)	7 (4.8)	39 (13.3)
20–34	385 (87.5)	135 (91.8)	250 (85.3)
> 34	9 (2.1)	5 (3.4)	4 (1.4)
**Residence**			
Urban	183 (41.6)	49 (33.3)	134 (45.7)
Rural	257 (58.4)	98 (66.7)	159 (54.3)
**Level of Education**			
Tertiary	75 (17.0)	20 (13.6)	55 (18.8)
Secondary	156 (35.5)	55 (37.4)	101 (34.5)
Primary	187 (42.5)	60 (40.8)	127 (43.3)
Uneducated	22 (5.0)	12 (8.2)	10 (3.4)
**Employment status**			
Employed	163 (37.1)	41 (27.9)	122 (41.6)
Unemployed	277 (62.9)	106 (72.1)	171 (58.4)
**Religion**			
Anglican	188 (42.7)	60 (40.8)	128 (43.7)
Catholic	137 (31.1)	47 (32.0)	90 (30.7)
Pentecostal	69 (15.7)	26 (17.7)	43 (14.7)
Moslem	46 (10.5)	14 (9.5)	32 (10.9)
**Marital status**			
Married	420 (95.4)	144 (98.0)	276 (94.2)
Unmarried	20 (4.6)	03 (2.0)	17 (5.8)
**Partner support**			
Yes	413 (93.9)	139 (94.6)	274 (93.5)
No	27 (6.1)	8 (5.4)	19 (6.5)
**Preferred sex of the baby**			
Yes	376 (85.5)	130 (88.4)	246 (84.0)
No	64 (14.5)	17 (11.6)	47 (16.0)

### Obstetric characteristics of study participants

Of the 440 participants, most were para 1–4 (88.2%), had used PPFP prior to the current pregnancy (67.5%) and had their pregnancies planned (57.2%) (Table 2). The majority (96.4%) of the participants had live births in the antecedent deliveries. The proportions of antecedent birth outcomes, use and time of initiation of PPFP, return of menses after delivery, and whether the pregnancy was planned differed significantly between mothers with short interbirth interval and those without short interbirth interval.

**Table 2:** Obstetric characteristics of women with antecedent caesarean delivery at Mbarara Regional Referral Hospital

**Variable**	**Total (*N* = 440), n (%)**	**Short interbirth interval, n (%)**	
		**Yes (*n* = 147)**	**No (*n* = 293)**
**Parity**			
1–4	388 (88.2)	126 (85.7)	262 (894)
≥ 5	52 (11.8)	21 (14.3)	31 (10.6)
**Antecedent birth outcome**			
Live birth	424 (96.4)	135 (91.8)	289 (98.6)
Still birth	16 (3.6)	12 (8.2)	4 (1.4)
**Desired more children**			
Yes	395 (89.8)	131 (89.1)	264 (90.1)
No	45 (10.2)	16 (10.9)	29 (9.9)
**Postnatal care attendance**^a^			
Yes	116 (26.4)	36 (24.5)	80 (27.3)
No	324 (73.6)	111(75.5)	213 (72.7)
**PPFP use**			
Yes	297 (67.5)	65 (44.2)	232 (79.2)
No	143 (32.5)	82 (55.8)	61 (20.8)
**Time at initiation of PPFP**			
< 6 weeks	80 (26.9)	22 (33.8)	58 (25)
6 weeks–1 year	159 (53.5)	38 (58.5)	121 (52.2)
> 1 year	58 (19.5)	5 (7.7)	53 (22.8)
**PPFP method used**			
Pills	15 (5.1)	4 (6.2)	11 (4.7)
Injectables	175 (58.9)	44 (67.7)	131 (56.5)
Implants	87 (29.3)	14 (21.5)	73 (31.5)
IUDs	14 (4.7)	1 (1.5)	13 (5.6)
Others	6 (2.0)	2 (3.1)	4 (1.7)
**Exclusive breastfeeding**			
≥ 6 months	295 (70.2)	87 (65.4)	208 (72.5)
< 6 months	125 (29.8)	46 (34.6)	79 (27.5)
**Resumption of coitus**			
≥ 1 month	420 (95.5)	138 (94.5)	282 (96.3)
< 1 month	20 (4.5)	8 (5.5)	11 (3.8)
**Return of menses**			
≥ 6 months	186 (42.3)	53 (36.1)	133(45.4)
< 6 months	254 (57.7)	94 (63.9)	160 (54.6)
**Planned current pregnancy**			
Planned	252 (57.3)	44 (29.9)	208 (71.0)
Unplanned	188 (42.7)	103 (70.1)	85 (29.0)

*PPFP* Postpartum Family Planning, *IUD* Intrauterine device, ^a^Postnatal care attendance considered was for the previous pregnancy

### Prevalence of short interbirth interval

Of the 440 participants who were enrolled into the study, 147 had short interbirth interval, for a prevalence of 33.4% (95% CI: 29.1–38.0). Among the 147 women with short interbirth interval, 34 (23%) had an interbirth interval of < 18 months, 43 (29.3%) had interbirth interval between 18–23 months, and the remaining 70 (47%) had interbirth interval of 24–32 months.

### Factors associated with short interbirth interval

At multivariable analysis, age less than 20 years (aPR = 0.25; 95%CI: 0.10–0.64, *P* = 0.004), history of having had a still birth at an antecedent pregnancy (aPR = 3.95; 95%CI: 1.43–10.9, *P* = 0.008), non-use of a postpartum family planning method (aPR = 2.24; 95%CI: 1.57–3.20, *P* < 0.001), and having an unplanned pregnancy (aPR = 3.59; 95%CI: 2.35–5.49, *P* < 0.001) were independently associated with a short interbirth interval (Table 3).

**Table 3:** Factors associated with short interbirth among women with antecedent caesarean deliveries at Mbarara Regional Referral Hospital

Variable	%Short IDI n/N (%)	Univariable analysis		Multivariable analysis	
		**cPR (95%CI)**	***P*-value**	**aPR (95%CI)**	***P*-value**
**Age in years**					
20–34	135/385 (35.1)	Ref		Ref	
< 20	7/46 (15.2)	0.38 (0.20–0.93)	0.031	0.25 (0.10–0.64)	0.004
> 34	5/9 (55.6)	1.58 (0.65–3.87)	0.312	1.08 (0.36–3.18)	0.892
**Residence**					
Urban	49/183 (26.8)	Ref		Ref	
Rural	98/257 (38.1)	1.42 (1.01 -2.01)	0.043	1.28 (0.85–1.93)	0.244
**Level of Education**					
Tertiary	20/75 (26.7)	Ref		Ref	
Secondary	55/156 (35.3)	1.32 (0.79–2.21)	0.285	1.30 (0.73–2.32)	0.374
Primary	60/187 (32.1)	1.20 (0.73–1.99)	0.474	0.89 (0.58–1.84)	0.974
Uneducated	12/22 (54.6)	2.05 (1.00–4.18)	0.050	1.08 (0.44–2.68)	0.859
**Employment status**					
Employed	41/163 (25.2)	Ref		Ref	
Unemployed	106/277 (38.3)	1.52 (1.06–2.18)	0.023	1.32 (0.84–2.06)	0.223
**Marital status**					
Married	144/420 (34.3)	Ref	Ref		
Unmarried	3/ 20(15.0)	0.44 (0.14–1.37)	0.156	0.26 (0.06–1.05)	0.059
**Antecedent birth outcome**					
Live birth	135/424 (31.8)	Ref		Ref	
Still birth	12/16 (75.0)	2.36 (1.31–4.24)	0.004	3.95 (1.43–10.9)	0.008
**PPFP use**					
Yes	65/297 (21.9)	Ref		Ref	
No	82/143 (57.3)	2.65 (1.89–3.63)	< 0.001	2.24 (1.57–3.20)	< 0.001
**Time of initiation of PPFP**^a^					
< 6 weeks	22/80 (27.5)	Ref			
6 weeks-1 year	42/159 (26.4)	0.96 (0.58–1.61)	0.878		
> 1 year	5/58 (8.6)	0.31 (0.12–0.83)	0.019		
**Exclusive breastfeeding**					
≥ 6 months	87/295 (29.5)	Ref		Ref	
< 6 months	46/125 (36.8)	1.24 (0.87–1.78)	0.225	1.14 (0.77–1.70)	0.506
**Return of menses**					
> 6 months	53/184 (28.8)	Ref		Ref	
< 6 months	94/250 (37.6)	1.39(0.93–1.84)	0.120	1.27 (0.88–1.82)	0.197
**Planned current pregnancy**					
Planned	44/252 (17.5)	Ref		Ref	
Unplanned	103/188 (54.8)	3.14 (2.20–4.47)	< 0.001	3.59 (2.35–5.49)	< 0.001

*PPFP* Postpartum Family Planning, *cPR* Crude Prevalence Ratio, *aPR* Adjusted Prevalence Ratio, *CI* Confidence Interval, *Ref* Reference category, ^a^Eliminated from multivariable model due to collinearity

## Discussion

Adequate interbirth intervals allow mothers to recover from the effects of pregnancy and to be in optimum health before the next pregnancy, by enabling replenishment of macro-and micronutrient stores that get depleted during pregnancy and lactation. This study determined the prevalence of short interbirth interval and associated factors among women who had previously delivered by cesarean section at a tertiary hospital in southwestern Uganda. One out of every three women with an antecedent cesarean delivery had a short interbirth interval in the current study. The prevalence of short interbirth interval was significantly higher among women with history of still births at previous pregnancies, those who did not use postpartum family planning methods, and those whose pregnancies were unplanned, compared to their counterparts. However, the prevalence of short interbirth interval was significantly lower among young mothers (< 20 years).

There is paucity of literature on prevalence of short interbirth intervals among women who have had previous cesarean section deliveries, making it difficult to compare our findings. Nonetheless, the prevalence in this study is higher than the reported worldwide prevalence of short interbirth intervals (25%) in the general population, and the regional prevalence in the sub-Saharan Africa (20%) [3].

In the current study, women who had still births at antecedent pregnancies were more likely to have short interbirth intervals compared to those who had live births. This is in agreement with previous findings from Bangladesh [17], Ethiopia [18, 19], and Uganda [20]. This finding may be attributed to the fact that couples with a previous still birth may intentionally deliver another baby to replace the lost child as early as possible. Mothers with infant and perinatal deaths also lack the protective mechanism of lactation, hence menses and fertility return early resulting in short interbirth intervals [14]. On the basis of this finding, women with still births and perinatal deaths should be targeted for family planning counseling to ensure optimum birth spacing.

In this study, non-use of postpartum family planning, and having unplanned pregnancy were associated with short interbirth intervals. This finding is consistent with findings from previous studies [6, 20–22]. Women who have unplanned pregnancies are likely not to be on family planning methods, and often rely on natural family planning methods, including lactational amenorrhea. In addition, women who plan their pregnancies may follow the recommendations for child spacing, such as use of postpartum family planning and hence end up with optimal birth intervals. Therefore, there is a need to counsel women and their spouses about contraception and opportunities for safer conception, given that pregnancy intentions are associated with having planned pregnancies [23].

Our study found that young mothers (< 20 years) were less likely to have short interbirth intervals compared to their older counterparts. Similar findings were reported in Bangladesh [24] and Ethiopia [18]. However, in USA, a study found that short interbirth intervals were common in teen pregnancies [25]. Similarly, our finding contradicts findings from a study done in selected sub-Saharan African countries, which reported that younger women tend to have shorter birth intervals [4]. The plausible explanation for this finding in our study, could be because teenage mothers believe they have more time and therefore are not under any pressure to deliver quickly, and may tend to wait longer [24]. Delivery by cesarean section is perceived negatively by most African women as not being “womanly” enough; this negative perception is more in teenage mothers [26]. The young mothers may therefore tend to wait longer in order to be given chance to deliver vaginally at the next delivery. Moreover, many of these young mothers may not be married at the time of their first pregnancy; marriage is known to influence interbirth interval [27]. However, a study in Uganda found that more than half of women who had their first birth < 18 years had repeat adolescent birth [28]. This may suggest that other socio-cultural factors, including male child preference, maternal age at marriage, and decision-making powers being vested in the husband, may also influence the decision to use a postpartum family planning method, hence affecting birth intervals [29–31].

Given the heightened risk for poor maternal and perinatal outcomes associated with sub-optimal child spacing among women with previous cesarean deliveries [8], our findings point towards the need to strengthen and scale-up child-spacing programs in the region, especially targeting women with cesarean deliveries. This may necessitate use of more innovative and multifaceted approaches to facilitate uptake of modern family planning methods including postpartum intrauterine devices (PPIUDs) that have proved to have expulsion rates that are low and comparable to interval IUD insertions in the region [32]. Strengthening prenatal and postnatal family planning counselling may also improve uptake of PPFP, and improve birth spacing [33]. Additionally, open dialogue sessions on birth spacing, organized by women's groups in communities, may also improve the uptake of modern family planning methods even further. As has been demonstrated elsewhere [34], such community mobilization approaches can result in positive changes in health behaviors, by encouraging active participatory learning Mothers who have had perinatal deaths and those with still births, should especially be targeted during postnatal family planning counselling sessions. Future studies are required to assess the implications short interbirth intervals, with regard to maternal and perinatal outcomes, among women with cesarean section deliveries in our Ugandan setting.

Our study is not without limitations. First, some data on dependent and independent variables were based on self-reports. This may have resulted into social-desirability bias [35]. Nevertheless, we verified these data using hospital medical forms and antenatal booking cards. Second, the cross-sectional nature of our study design limits us from making causal inferences from our findings. The strength of our study lies in it being one of initial studies to characterize birth intervals in the unique population of women with antecedent cesarean deliveries, as most earlier studies have heavily focused on the general population of women of child-bearing age.

## Conclusion

This study found that one out of every three women who delivered at MRRH in southwestern Uganda, with an antecedent cesarean delivery had a short interbirth interval. Short interbirth interval was significantly more common among women with history of still births at previous pregnancies, those who did not use postpartum family planning methods, and those whose pregnancies were unplanned, compared to their counterparts. Young mothers (< 20 years) were less likely to have short interbirth intervals compared to those who were 20 years or older. Efforts should be made to strengthen and scale up child-spacing programs targeting women with previous cesarean deliveries, given the high frequency of short interbirth intervals in this study population.

## Data Availability

The datasets generated and analysed for this study are available from the corresponding author, upon request.

## Abbreviations

aPR: Adjusted Prevalence Ratio
CI: Confidence Interval
cPR: Crude Prevalence Ratio
DRC: Democratic Republic of Congo
IUD: Intrauterine Device
MRRH: Mbarara Regional Referral Hospital
PPFP: Postpartum Family Planning
PPIUD: Postpartum Intrauterine Device
PR: Prevalence Ratio
WHO: World Health Organization
